# (*Z*)-3-[1-(4-Methoxy­anilino)ethyl­idene]-4,5-dihydro­furan-2(3*H*)-one

**DOI:** 10.1107/S1600536808016504

**Published:** 2008-06-21

**Authors:** Li-Ping Zhang, Cai-Hua Ni, Zhan-Hui Zhang

**Affiliations:** a School of Chemical and Materials Engineering, Jiangnan University, 1800 Lihu Road, Wuxi 214122, Jiangsu, People’s Republic of China; b School of Chemistry and Materials Science, Hebei Normal University, 113 Yuhua Road, Shijiazhuang 050000, Hebei, People’s Republic of China

## Abstract

In the title compound, C_13_H_15_NO_3_, the dihydro­furan­one ring is planar to within 0.012 (4) Å and it forms a dihedral angle of 42.8 (2)° with the benzene ring. The amino­ethyl­idene group is coplanar with the dihydro­furan­one ring. The meth­oxy group is slightly twisted away from the benzene ring. An intra­molecular N—H⋯O hydrogen bond, generating an *S*(6) ring, is observed. In the crystal structure, the mol­ecules exist as C—H⋯O hydrogen-bonded dimers.

## Related literature

For general background, see: Bartoli *et al.* (1994 ▶); Cimarelli & Palmieri (1996 ▶); Cimarelli *et al.* (1994 ▶); Elassar & El-Khair (2003 ▶); Greenhill (1977 ▶); Lubell *et al.* (1991 ▶); Michael *et al.* (1999 ▶); Negri *et al.* (2004 ▶); Reddy *et al.* (2005 ▶); Zhang *et al.* (2006 ▶).
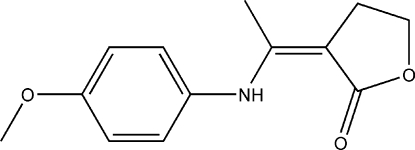


## Experimental

### 

#### Crystal data

C_13_H_15_NO_3_
*M* *_r_* = 233.26
Orthorhombic, 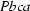

*a* = 12.562 (9) Å
*b* = 7.568 (5) Å
*c* = 24.531 (18) Å
*V* = 2332 (3) Å^3^
*Z* = 8
Mo *K*α radiation
μ = 0.10 mm^−1^
*T* = 293 (2) K
0.26 × 0.20 × 0.10 mm

#### Data collection

Bruker SMART CCD area-detector diffractometer
Absorption correction: multi-scan (*SADABS*; Sheldrick, 1996 ▶) *T*_min_ = 0.963, *T*_max_ = 0.990
8990 measured reflections
2051 independent reflections
1409 reflections with *I* > 2σ(*I*)
*R*_int_ = 0.051

#### Refinement

*R*[*F* ^2^ > 2σ(*F* ^2^)] = 0.064
*wR*(*F* ^2^) = 0.140
*S* = 1.20
2051 reflections
156 parameters
H-atom parameters constrained
Δρ_max_ = 0.17 e Å^−3^
Δρ_min_ = −0.21 e Å^−3^

### 

Data collection: *SMART* (Bruker, 1998 ▶); cell refinement: *SAINT* (Bruker, 1999 ▶); data reduction: *SAINT*; program(s) used to solve structure: *SHELXS97* (Sheldrick, 2008 ▶); program(s) used to refine structure: *SHELXL97* (Sheldrick, 2008 ▶); molecular graphics: *SHELXTL* (Sheldrick, 2008 ▶); software used to prepare material for publication: *SHELXTL*.

## Supplementary Material

Crystal structure: contains datablocks global, I. DOI: 10.1107/S1600536808016504/ci2605sup1.cif

Structure factors: contains datablocks I. DOI: 10.1107/S1600536808016504/ci2605Isup2.hkl

Additional supplementary materials:  crystallographic information; 3D view; checkCIF report

## Figures and Tables

**Table 1:** Hydrogen-bond geometry (Å, °)

*D*—H⋯*A*	*D*—H	H⋯*A*	*D*⋯*A*	*D*—H⋯*A*
N1—H1⋯O2	0.86	2.13	2.762 (4)	130
C6—H6⋯O2^i^	0.93	2.53	3.405 (4)	158

Symmetry code: (i) 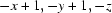.

**Fig. 1.**
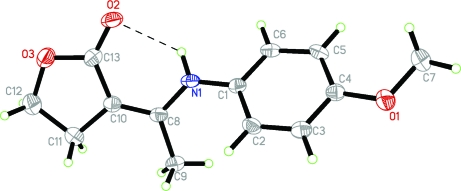
The molecular structure of the title compound, showing 30% probability displacement ellipsoids. The dashed line indicates a hydrogen bond.

**Fig. 2.**
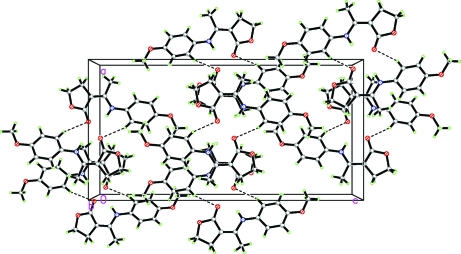
The crystal packing of the title compound, showing C—H···O hydrogen-bonded dimers.

## supplementary crystallographic information

### Comment

β-Enamino esters are a highly versatile class of intermediates for the synthesis of heterocycles (Reddy *et al.*, 2005; Negri *et al.*, 2004) and biologically active compounds, such as β-enamino acids, γ-enamino alcohols or β-enamino esters (Lubell *et al.*, 1991; Bartoli *et al.*, 1994; Cimarelli *et al.*, 1994; Cimarelli & Palmieri, 1996). Many synthetic methods have been developed for the preparation of these compounds (Greenhill, 1977; Michael *et al.*, 1999; Elassar & El-Khair, 2003). We synthesized a class of β-enamino compounds by reacting β-dicarbonyl compounds with amines in the presence of a catalytic amount of indium tribromide (Zhang *et al.* 2006). We report herein the crystal structure of the title compound (Fig.1).

In the title molecule, the dihydrofuranone ring is planar to within ±0.012 (4) Å and it forms a dihedral angle of 42.8 (2)° with the benzene ring. The aminoethylidene group is coplanar with the dihydrofuranone ring. The methoxy group is slightly twisted away from the benzene ring, with a C7—O1—C4—C5 torsion angle of 5.9 (5)°. The C11—C12 bond length [1.561 (5) Å] is markedly longther than usual C—C bond length. The N1—C8 bond length [1.400 (4) Å] is slightly shorter than the N1—C1 [1.439 (4) Å] bond length, indicating a weak electron delocalization. An intramolecular N1—H1···O2 hydrogen bond generating an S(6) ring is observed.

In the crystal structure, intermolecular C6—H6···O2 hydrogen bonds create centrosymmetric hydrogen-bonded dimers (Fig.2).

### Experimental

A mixture of the 2-acetylcyclobutanone (5 mmol), 4-methoxybenzenamine (5 mmol) and InBr_3_ (0.05 mmol) was stirred at room temperature for 1 h. After completion of the reaction, the reaction mixture was diluted with H_2_O (10 ml) and extracted with EtOAc (210 ml). The combined organic layers were dried, concentrated, purified by column chromatography on SiO_2_ with ethyl acetate-cyclohexane (1:8). Pale yellow solid was obtained with a yield of 89% (m.p. 339–341 K). IR (neat):ν 3526, 2976, 1683, 1628, 1513, 1475, 1228, 1114, 1029, 947, 822, 763 cm^-1^; ^1^H NMR(CDCl_3_, 300 MHz): δ 1.91 (s, 3H), 2.89 (t, 2H), 4.02 (s, 3H), 4.34 (t, 2H), 6.84 (d, 1H), 6.99 (d, 1H), 9.77 (br s, 1H, NH). ^13^C NMR(CDCl_3_, 75 MHz): δ 17.4, 26.4, 63.7, 65.2, 87.6, 114.8, 126.6, 131.8, 154.6, 156.8, 173.9. ESI-MS: 233(*M*+1)^+^. Analysis calculated for C_13_H_15_NO: C 66.94, H 6.48, N 6.00%; found: C 67.17, H 5.58, N 5.68%. Single crystals suitable for X-ray diffraction study were obtained from ethyl acetate-cyclohexane by slow evaporation at room temperature.

### Refinement

H atoms were placed in geometrically idealized positions and constrained to ride on their parent atoms, with N-H = 0.86 Å, C-H = 0.93–0.97 Å, and *U*_iso_(H) = 1.5*U*_eq_(CH_3_)or 1.2*U*_eq_(C,N). Each methyl group was allowed to rotate freely about its C—C bond.

### Crystal data

**Table:**

C_13_H_15_NO_3_	*F*_000_ = 992
*M**_r_* = 233.26	*D*_x_ = 1.329 Mg m^−^^3^*D*_m_ = 1.329 Mg m^−^^3^*D*_m_ measured by not measured
Orthorhombic, *P**b**c**a*	Mo *K*α radiation λ = 0.71073 Å
Hall symbol: -P 2ac 2ab	Cell parameters from 2424 reflections
*a* = 12.562 (9) Å	θ = 2.3–26.5º
*b* = 7.568 (5) Å	µ = 0.10 mm^−^^1^
*c* = 24.531 (18) Å	*T* = 293 (2) K
*V* = 2332 (3) Å^3^	Block, yellow
*Z* = 8	0.26 × 0.20 × 0.10 mm

### Data collection

**Table:**

Bruker SMART CCD area-detector diffractometer	2051 independent reflections
Radiation source: fine-focus sealed tube	1409 reflections with *I* > 2σ(*I*)
Monochromator: graphite	*R*_int_ = 0.051
*T* = 293(2) K	θ_max_ = 25.0º
φ and ω scans	θ_min_ = 1.7º
Absorption correction: multi-scan(SADABS; Sheldrick, 1996)	*h* = −6→14
*T*_min_ = 0.963, *T*_max_ = 0.990	*k* = −6→9
8990 measured reflections	*l* = −29→27

### Refinement

**Table:**

Refinement on *F*^2^	Secondary atom site location: difference Fourier map
Least-squares matrix: full	Hydrogen site location: inferred from neighbouring sites
*R*[*F*^2^ > 2σ(*F*^2^)] = 0.064	H-atom parameters constrained
*wR*(*F*^2^) = 0.140	*w* = 1/[σ^2^(*F*_o_^2^) + (0.0345*P*)^2^ + 1.787*P*] where *P* = (*F*_o_^2^ + 2*F*_c_^2^)/3
*S* = 1.20	(Δ/σ)_max_ = 0.001
2051 reflections	Δρ_max_ = 0.18 e Å^−^^3^
156 parameters	Δρ_min_ = −0.21 e Å^−^^3^
Primary atom site location: structure-invariant direct methods	Extinction correction: none

### Special details

**Table:**

Geometry. All e.s.d.'s (except the e.s.d. in the dihedral angle between two l.s. planes) are estimated using the full covariance matrix. The cell e.s.d.'s are taken into account individually in the estimation of e.s.d.'s in distances, angles and torsion angles; correlations between e.s.d.'s in cell parameters are only used when they are defined by crystal symmetry. An approximate (isotropic) treatment of cell e.s.d.'s is used for estimating e.s.d.'s involving l.s. planes.
Refinement. Refinement of *F*^2^ against ALL reflections. The weighted *R*-factor *wR* and goodness of fit *S* are based on *F*^2^, conventional *R*-factors *R* are based on *F*, with *F* set to zero for negative *F*^2^. The threshold expression of *F*^2^ > σ(*F*^2^) is used only for calculating *R*-factors(gt) *etc*. and is not relevant to the choice of reflections for refinement. *R*-factors based on *F*^2^ are statistically about twice as large as those based on *F*, and *R*- factors based on ALL data will be even larger.

### Fractional atomic coordinates and isotropic or equivalent isotropic displacement parameters (Å^2^)

**Table:**

	*x*	*y*	*z*	*U*_iso_*/*U*_eq_	
O1	0.60436 (17)	0.4248 (3)	0.28822 (9)	0.0584 (7)	
O2	0.56144 (16)	0.3225 (3)	−0.03039 (9)	0.0577 (7)	
O3	0.67421 (17)	0.2671 (3)	−0.09776 (9)	0.0588 (7)	
N1	0.66238 (18)	0.4237 (3)	0.06464 (10)	0.0458 (7)	
H1	0.6053	0.4365	0.0457	0.055*	
C1	0.6483 (2)	0.4258 (4)	0.12125 (12)	0.0359 (7)	
C2	0.7161 (2)	0.3370 (4)	0.15658 (12)	0.0418 (7)	
H2	0.7736	0.2740	0.1427	0.050*	
C3	0.6988 (2)	0.3416 (4)	0.21161 (12)	0.0445 (8)	
H3	0.7454	0.2832	0.2349	0.053*	
C4	0.6133 (2)	0.4313 (4)	0.23285 (12)	0.0397 (7)	
C5	0.5438 (2)	0.5155 (4)	0.19836 (12)	0.0408 (7)	
H5	0.4845	0.5739	0.2122	0.049*	
C6	0.5626 (2)	0.5127 (4)	0.14287 (12)	0.0402 (7)	
H6	0.5159	0.5712	0.1196	0.048*	
C7	0.5122 (3)	0.4963 (5)	0.31207 (14)	0.0639 (10)	
H7A	0.5103	0.6214	0.3058	0.096*	
H7B	0.5129	0.4738	0.3506	0.096*	
H7C	0.4504	0.4424	0.2961	0.096*	
C8	0.7527 (2)	0.4043 (4)	0.03547 (11)	0.0379 (7)	
C9	0.8577 (2)	0.4487 (4)	0.06065 (13)	0.0474 (8)	
H9A	0.8960	0.3417	0.0683	0.071*	
H9B	0.8464	0.5128	0.0939	0.071*	
H9C	0.8983	0.5201	0.0358	0.071*	
C10	0.7493 (2)	0.3554 (4)	−0.01772 (11)	0.0398 (7)	
C11	0.8409 (2)	0.3275 (5)	−0.05534 (12)	0.0511 (8)	
H11A	0.8877	0.2350	−0.0421	0.061*	
H11B	0.8816	0.4353	−0.0601	0.061*	
C12	0.7864 (3)	0.2733 (5)	−0.10790 (14)	0.0625 (10)	
H12A	0.8019	0.3582	−0.1365	0.075*	
H12B	0.8118	0.1582	−0.1195	0.075*	
C13	0.6529 (3)	0.3163 (4)	−0.04569 (12)	0.0450 (8)	

### Atomic displacement parameters (Å^2^)

**Table:**

	*U*^11^	*U*^22^	*U*^33^	*U*^12^	*U*^13^	*U*^23^
O1	0.0501 (14)	0.0825 (18)	0.0428 (14)	0.0102 (13)	0.0055 (11)	0.0044 (12)
O2	0.0335 (12)	0.0875 (18)	0.0520 (14)	−0.0095 (12)	−0.0071 (10)	−0.0009 (12)
O3	0.0559 (14)	0.0769 (17)	0.0435 (13)	−0.0083 (13)	−0.0030 (11)	−0.0100 (11)
N1	0.0240 (13)	0.0681 (18)	0.0452 (16)	0.0017 (12)	−0.0050 (11)	−0.0001 (13)
C1	0.0293 (15)	0.0367 (16)	0.0416 (17)	−0.0034 (13)	−0.0038 (13)	−0.0004 (13)
C2	0.0341 (16)	0.0391 (17)	0.0522 (19)	0.0082 (14)	0.0008 (14)	−0.0047 (14)
C3	0.0389 (18)	0.0450 (19)	0.050 (2)	0.0052 (15)	−0.0065 (14)	0.0042 (15)
C4	0.0347 (16)	0.0399 (17)	0.0444 (19)	−0.0048 (14)	0.0002 (13)	0.0017 (13)
C5	0.0265 (15)	0.0430 (18)	0.0528 (19)	0.0057 (13)	0.0053 (14)	0.0017 (14)
C6	0.0262 (15)	0.0436 (18)	0.0510 (19)	0.0019 (13)	−0.0058 (13)	0.0071 (14)
C7	0.054 (2)	0.085 (3)	0.053 (2)	0.000 (2)	0.0131 (17)	−0.0049 (19)
C8	0.0307 (15)	0.0367 (16)	0.0463 (18)	0.0035 (13)	−0.0058 (14)	0.0018 (13)
C9	0.0352 (17)	0.055 (2)	0.052 (2)	−0.0022 (15)	−0.0074 (14)	−0.0030 (15)
C10	0.0354 (16)	0.0401 (17)	0.0438 (18)	−0.0025 (14)	0.0001 (14)	0.0017 (14)
C11	0.0454 (19)	0.054 (2)	0.054 (2)	0.0036 (16)	0.0042 (16)	−0.0014 (16)
C12	0.061 (2)	0.072 (3)	0.055 (2)	0.001 (2)	0.0083 (18)	−0.0076 (18)
C13	0.045 (2)	0.0480 (19)	0.0417 (19)	−0.0052 (16)	−0.0050 (15)	0.0015 (14)

### Geometric parameters (Å, °)

**Table:**

O1—C4	1.364 (4)	C6—H6	0.93
O1—C7	1.406 (4)	C7—H7A	0.96
O2—C13	1.209 (4)	C7—H7B	0.96
O3—C13	1.357 (4)	C7—H7C	0.96
O3—C12	1.432 (4)	C8—C10	1.357 (4)
N1—C8	1.349 (4)	C8—C9	1.495 (4)
N1—C1	1.400 (4)	C9—H9A	0.96
N1—H1	0.86	C9—H9B	0.96
C1—C6	1.368 (4)	C9—H9C	0.96
C1—C2	1.389 (4)	C10—C13	1.424 (4)
C2—C3	1.368 (4)	C10—C11	1.490 (4)
C2—H2	0.93	C11—C12	1.516 (4)
C3—C4	1.374 (4)	C11—H11A	0.97
C3—H3	0.93	C11—H11B	0.97
C4—C5	1.372 (4)	C12—H12A	0.97
C5—C6	1.382 (4)	C12—H12B	0.97
C5—H5	0.93		
			
C4—O1—C7	117.9 (3)	H7B—C7—H7C	109.5
C13—O3—C12	110.4 (2)	N1—C8—C10	120.9 (3)
C8—N1—C1	129.3 (2)	N1—C8—C9	119.9 (3)
C8—N1—H1	115.3	C10—C8—C9	119.1 (3)
C1—N1—H1	115.3	C8—C9—H9A	109.5
C6—C1—C2	118.2 (3)	C8—C9—H9B	109.5
C6—C1—N1	119.3 (3)	H9A—C9—H9B	109.5
C2—C1—N1	122.4 (3)	C8—C9—H9C	109.5
C3—C2—C1	120.4 (3)	H9A—C9—H9C	109.5
C3—C2—H2	119.8	H9B—C9—H9C	109.5
C1—C2—H2	119.8	C8—C10—C13	123.1 (3)
C2—C3—C4	120.8 (3)	C8—C10—C11	127.6 (3)
C2—C3—H3	119.6	C13—C10—C11	109.2 (3)
C4—C3—H3	119.6	C10—C11—C12	102.5 (3)
O1—C4—C5	125.3 (3)	C10—C11—H11A	111.3
O1—C4—C3	115.1 (3)	C12—C11—H11A	111.3
C5—C4—C3	119.5 (3)	C10—C11—H11B	111.3
C4—C5—C6	119.4 (3)	C12—C11—H11B	111.3
C4—C5—H5	120.3	H11A—C11—H11B	109.2
C6—C5—H5	120.3	O3—C12—C11	107.8 (3)
C1—C6—C5	121.6 (3)	O3—C12—H12A	110.1
C1—C6—H6	119.2	C11—C12—H12A	110.1
C5—C6—H6	119.2	O3—C12—H12B	110.1
O1—C7—H7A	109.5	C11—C12—H12B	110.1
O1—C7—H7B	109.5	H12A—C12—H12B	108.5
H7A—C7—H7B	109.5	O2—C13—O3	119.4 (3)
O1—C7—H7C	109.5	O2—C13—C10	130.6 (3)
H7A—C7—H7C	109.5	O3—C13—C10	110.0 (3)
			
C8—N1—C1—C6	154.5 (3)	C1—N1—C8—C9	−23.1 (5)
C8—N1—C1—C2	−28.0 (5)	N1—C8—C10—C13	−2.0 (4)
C6—C1—C2—C3	−2.0 (4)	C9—C8—C10—C13	−177.9 (3)
N1—C1—C2—C3	−179.5 (3)	N1—C8—C10—C11	−179.7 (3)
C1—C2—C3—C4	1.1 (5)	C9—C8—C10—C11	4.4 (5)
C7—O1—C4—C5	5.9 (5)	C8—C10—C11—C12	179.4 (3)
C7—O1—C4—C3	−172.8 (3)	C13—C10—C11—C12	1.4 (3)
C2—C3—C4—O1	179.5 (3)	C13—O3—C12—C11	1.9 (4)
C2—C3—C4—C5	0.8 (5)	C10—C11—C12—O3	−1.9 (4)
O1—C4—C5—C6	179.6 (3)	C12—O3—C13—O2	178.4 (3)
C3—C4—C5—C6	−1.8 (4)	C12—O3—C13—C10	−1.0 (4)
C2—C1—C6—C5	1.0 (4)	C8—C10—C13—O2	2.3 (5)
N1—C1—C6—C5	178.5 (3)	C11—C10—C13—O2	−179.6 (3)
C4—C5—C6—C1	0.9 (4)	C8—C10—C13—O3	−178.5 (3)
C1—N1—C8—C10	161.0 (3)	C11—C10—C13—O3	−0.4 (3)

### Hydrogen-bond geometry (Å, °)

**Table:**

*D*—H···*A*	*D*—H	H···*A*	*D*···*A*	*D*—H···*A*
N1—H1···O2	0.86	2.13	2.762 (4)	130
C6—H6···O2^i^	0.93	2.53	3.405 (4)	158

Symmetry codes: (i) −*x*+1, −*y*+1, −*z*.
